# Estimation Statistics, One Year Later

**DOI:** 10.1523/ENEURO.0091-21.2021

**Published:** 2021-03-31

**Authors:** Christophe Bernard

Where are we now, 1 year after *eNeuro* launched an initiative to encourage the use of estimation statistics in neuroscience? We started to attract attention to estimation statistics in August 2019 with an editorial (Bernard, 2019) and opinion paper (Calin-Jageman and Cumming, 2019). In February 2020, I asked Reviewing Editors to identify papers that could benefit from converting to estimation statistics. One hundred were thus identified. And, overall in 2020, 52 published papers included estimation statistics in their analyses! One of the earliest papers to include estimation statistics was from my own laboratory because I was determined to be sure for myself that this was a feasible step to take.

So, thank you all for making this initiative a success. Using estimation statistics, in addition to the classical significance testing (and the *p* value), is not a constraint. The feedback we get from authors who did it is nothing but positive. The display of results is much clearer, and the effect sizes can be seen directly; the *p* value even becomes non-necessary in some instances. You may have noticed that we highlighted some of these papers throughout the past year, along with authors’ comments about their thoughts using estimation statistics; you can read their comments in a recent blog post (https://blog.eneuro.org/2021/02/discussion-est-stats-author-feedback).

Let us briefly recall what estimation statistics is and what its benefits are.

It is now clear for my own research that estimation statistics is more informative and more powerful to analyze and interpret my data. If I need to compare two conditions (e.g., the effect of a treatment on seizure frequency), I calculate the mean difference and the confidence interval. I can then estimate the effect size (basically, how far the mean difference is from the 0 value and how small is the confidence interval). There are three main advantages for presenting effect sizes. First, interpreting one’s data in terms of the precision and magnitude of an effect is more useful and more instructive. Your readers can see at once how justified your interpretation is. The metric is standardized and can be understood by all. Second, prior knowledge of effect sizes is important when designing a new study (e.g., for a power analysis). Third, effect sizes are particularly suited when multiple studies need to be compared (e.g., the efficiency of different COVID-19 vaccines).

With the reproducibility crisis, I became highly suspicious when reading in papers *p* < 0.05, *n* = 5 (note that in many papers, there is something magic with the *n* = 5 number of experiments to show something). Many papers have been published criticizing null hypothesis significance testing and the “dictatorship” of the *p* < 0.05 value; for example, see Singh Chawla (2017) and Gelman (2018). Many conclusions/interpretations published in top-tier journals were found to be overconfident because, despite a *p* value <0.05, there was no assurance of a meaningful and reproducible effect. The *p* value is nothing more than a probability, and the probability that there is no effect can be high. So, *p* can be <0.05 even if the study gives almost no information about the magnitude of the effect or how to try to replicate it. Perhaps, every new student in neuroscience should start watching the dance of the *p* values (https://youtu.be/5OL1RqHrZQ8) before doing any data analysis and interpretation. The video brilliantly shows how different samplings of two distributions can produce significant and nonsignificant results. You can explore the dance for yourself with an interactive tool on the New Statistics website (https://www.esci.thenewstatistics.com/esci-dances.html), which is great for teaching.

Overreliance on the *p* value can be blinding. You may have a *p* value <0.05, but your confidence in your interpretation can only be obtained when you report the effect size. In the same line, there is a tendency to say the lower the *p* value, the better. I always smile when I see what I call homeopathic *p* values reported in papers [i.e., *p* values lower than the maximum dilution (1/6 10^−23^), usually when you crunch big numbers].

However, it is important to keep in mind that estimation statistics and significance testing can complement each other. They provide different types of information. One just needs to know how to use these techniques and be aware of their limitations.

Using estimation statistics is straightforward and easy, which may explain why many of you started to use it in *eNeuro*. A good place to start is https://www.estimationstats.com/#/. There is a tutorial, and you can directly upload your datasets, and get the results and the figures. And it is free. There are also packages for R and for popular open-source statistical programs (https://thenewstatistics.com/itns/esci/jesci/).

Just have a go, and I am sure that you will be convinced by the power of this approach.

On this issue, my confidence interval is excellent and the effect size very large.
